# Supplementary material for: Trichoderma brevicompactum 6311: Prevention and Control of Phytophthora capsici and Its Growth-Promoting Effect
Source: J Fungi (Basel). 2025 Jan 30;11(2):105. doi: 10.3390/jof11020105 (PMC11856043; doi:10.3390/jof11020105)
Supplement: Supplementary file 1 [file jof-11-00105-s001.zip › Figure S1-S3.pdf]

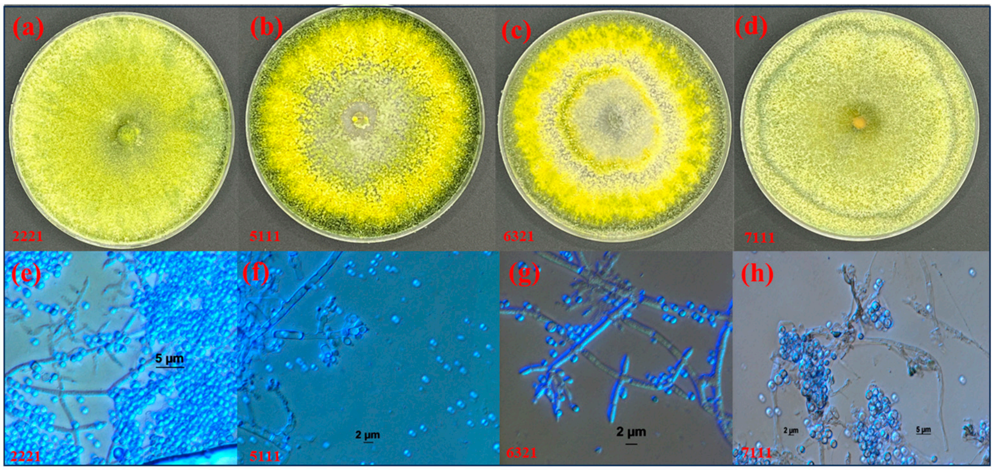

Figure S1. Colony morphology of four biocontrol microorganisms. (a) *Trichoderma* 2221; (b) *Trichoderma* 5111; (c) *Trichoderma* 6321; (d) *Trichoderma* 7111; spore morphology of four biocontrol microorganisms. (e) *Trichoderma* 2221; (f) *Trichoderma* 5111; (g) *Trichoderma* 6321; (h) *Trichoderma* 7111

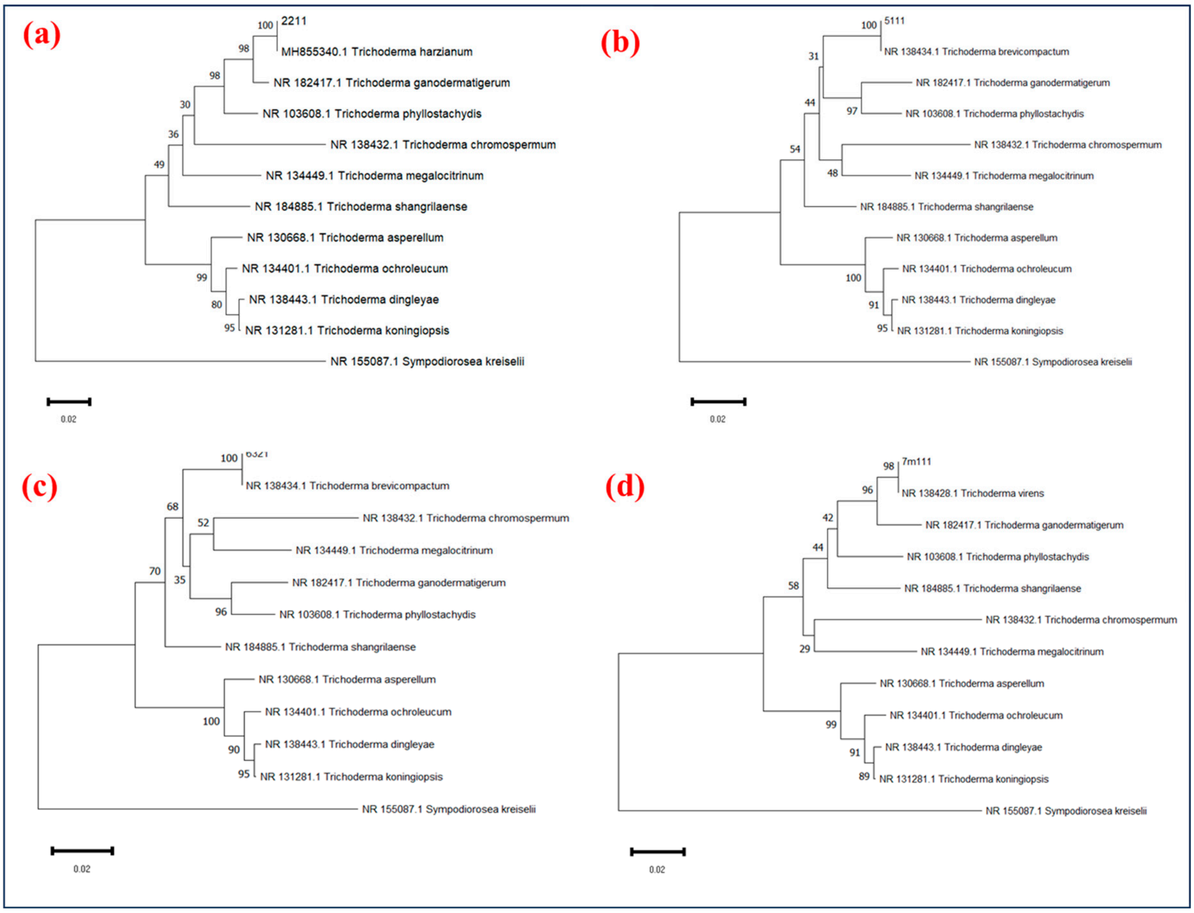

Figure S2. Molecular identification of four biocontrol microorganisms based on ITS. (a) *Trichoderma* 2221 developmental trees; (b) *Trichoderma* 5111 developmental trees; (c) *Trichoderma* 6321 developmental trees; (d) *Trichoderma* 7111 developmental trees.

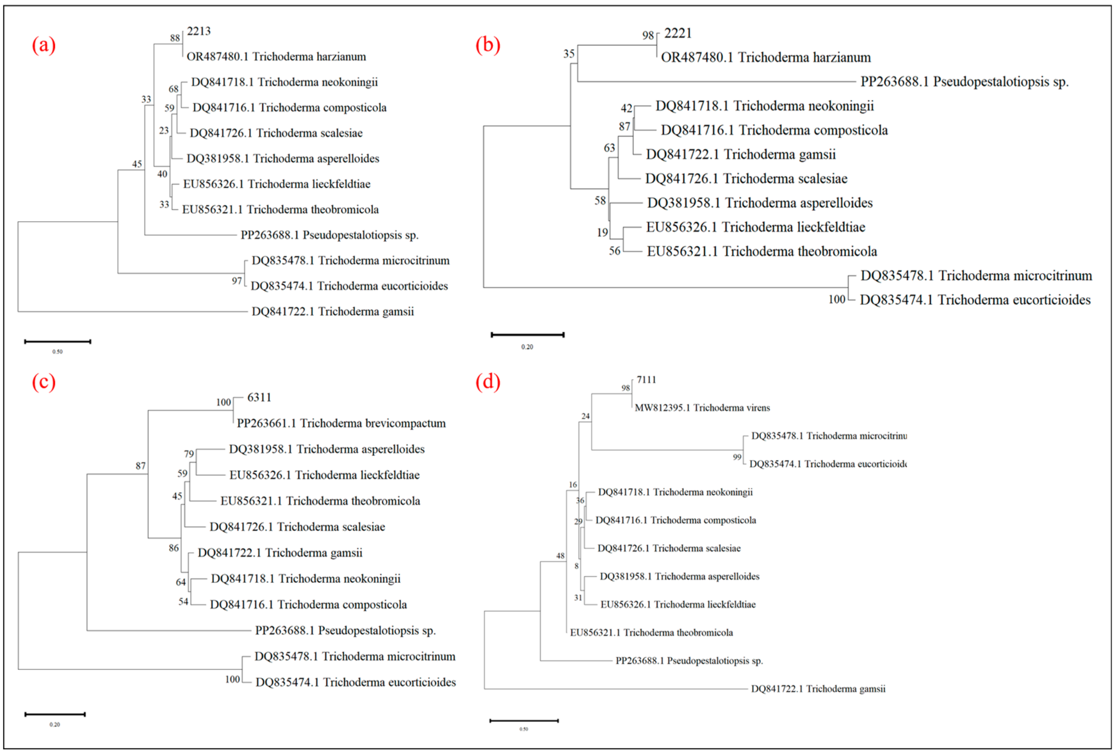

Figure S3. Molecular identification of four biocontrol microorganisms based on *tef1* primers. (a) *Trichoderma* 2213 developmental trees; (b) *Trichoderma* 2221 developmental trees; (c) *Trichoderma* 6311 developmental trees; (d) *Trichoderma* 7111 developmental trees.
