# Supplementary material for: Trichoderma brevicompactum 6311: Prevention and Control of Phytophthora capsici and Its Growth-Promoting Effect
Source: J Fungi (Basel). 2025 Jan 30;11(2):105. doi: 10.3390/jof11020105 (PMC11856043; doi:10.3390/jof11020105)
Supplement: Supplementary file 1 [file jof-11-00105-s001.zip › Table S1-Table S2.pdf]

Table S1 The sequencing data.

Sample	Raw Reads No	Raw Bases(bp)	Q30(bp)	GC(%)	N(%)	Q20(%)	Q30(%)
CK_1	44516792	6.72E+09	6.5E+09	41.95	0.004128	98.89	96.73
CK_2	40694632	6.14E+09	5.94E+09	41.96	0.004413	98.88	96.7
CK_3	43182466	6.52E+09	6.31E+09	41.96	0.00443	98.87	96.7
C2213_1	49009022	7.4E+09	7.18E+09	41.89	0.004442	98.98	96.96
C2213_2	48392974	7.31E+09	7.07E+09	41.97	0.004418	98.89	96.69
C2213_3	53903098	8.14E+09	7.89E+09	41.97	0.004559	98.96	96.91
C6311_1	37384848	5.65E+09	5.46E+09	42.27	0.004466	98.89	96.75
C6311_2	38869950	5.87E+09	5.69E+09	42.08	0.004411	98.96	96.93
C6311_3	43931210	6.63E+09	6.42E+09	42.31	0.004429	98.91	96.8

Table S2 The alignment information.

Sample	Clean Reads	Total Mapped	Multiple Mapped	Uniquely Mapped	Map Events	Mapped to Gene	Mapped to InterGene	Mapped to Exon
CK_1	43914048	36346564 (82.77%)	1255756 (3.45%)	35090808 (96.55%)	35090808	25930194 (73.89%)	9160614 (26.11%)	24942282 (96.19%)
CK_2	40154362	32484584 (80.90%)	994291 (3.06%)	31490293 (96.94%)	31490293	23039693 (73.16%)	8450600 (26.84%)	22119160 (96.00%)
CK_3	42558638	35055462 (82.37%)	1120648 (3.20%)	33934814 (96.80%)	33934814	24793926 (73.06%)	9140888 (26.94%)	23779750 (95.91%)
C2213_1	48377758	40635585 (84.00%)	1125827 (2.77%)	39509758 (97.23%)	39509758	28698070 (72.64%)	10811688 (27.36%)	27462159 (95.69%)
C2213_2	47706420	39827966 (83.49%)	1087433 (2.73%)	38740533 (97.27%)	38740533	28190976 (72.77%)	10549557 (27.23%)	26964862 (95.65%)
C2213_3	53147370	44266224 (83.29%)	1225038 (2.77%)	43041186 (97.23%)	43041186	31208331 (72.51%)	11832855 (27.49%)	29801866 (95.49%)
C6311_1	36862112	28766788 (78.04%)	824831 (2.87%)	27941957 (97.13%)	27941957	20364263 (72.88%)	7577694 (27.12%)	19596487 (96.23%)
C6311_2	38362190	31523464 (82.17%)	884543 (2.81%)	30638921 (97.19%)	30638921	22526336 (73.52%)	8112585 (26.48%)	21698234 (96.32%)
C6311_3	43315846	35018937 (80.85%)	1052085 (3.00%)	33966852 (97.00%)	33966852	25317904 (74.54%)	8648948 (25.46%)	24492040 (96.74%)
